# Neighborhood Walkability Among Older Adults With and Without Physical Disabilities

**DOI:** 10.1093/geroni/igaa057.960

**Published:** 2020-12-16

**Authors:** Rie Suzuki, Jennifer Blackwood, Noah Webster

**Affiliations:** 1 University of Michigan--Flint, Flint, Michigan, United States; 2 University of Michigan-Flint, Flint, Michigan, United States; 3 University of Michigan, Ann Arbor, Michigan, United States

## Abstract

Older adults with physical disabilities (PDs) often experience obstacles to walking locally. Although health promotion programs targeting physical activity are available in lower income, few studies have compared the walking experiences of older adults in these communities who have PDs with those who do not. The purpose of this study was to compare perceptions of neighborhood walkability among adults living in lower income communities with and without PDs. Participants (N=132) were recruited in 2018 at a regional health clinic in Flint, MI. To be eligible, participants had to be over 65 years old and Flint residents. A subsample (N=12) were then followed up with in 2019/2020. We defined PDs as having difficulty performing one or more activities of daily living. Descriptive statistics and analysis of covariance (ANCOVA) were performed. Of the 132 participants, the mean age in 2018 was 69.75 (SD=5.00). The majority were female (68%); African American (80%); single, divorced, or widowed (80%); and educated below GED level (84%). Older adults with PDs were less likely than those without to visit stores within walking distance and walk in their neighborhoods, and more likely to complain about a lot of traffic along the street. Analysis of the longitudinal data show that older adults who had PDs at time 1 were more likely at time 2 to 1) state that their neighborhoods were unsafe; and 2) perceive their neighborhoods more negatively. Findings suggest it is essential to develop disability-friendly support systems and accommodations to encourage walking in lower income communities.

